# Test Expectation Enhances Memory Consolidation across Both Sleep and Wake

**DOI:** 10.1371/journal.pone.0165141

**Published:** 2016-10-19

**Authors:** Erin J. Wamsley, Kelly Hamilton, Yvette Graveline, Stephanie Manceor, Elaine Parr

**Affiliations:** 1 Furman University, Department of Psychology and Program in Neuroscience, Greenville, South Carolina, United States of America; 2 Current Address: University of British Columbia, Department of Psychology, Vancouver, British Columbia, Canada; Universite de Lyon, FRANCE

## Abstract

Memory consolidation benefits from post-training sleep. However, recent studies suggest that sleep does not uniformly benefit all memory, but instead prioritizes information that is important to the individual. Here, we examined the effect of test expectation on memory consolidation across sleep and wakefulness. Following reports that information with strong “future relevance” is preferentially consolidated during sleep, we hypothesized that test expectation would enhance memory consolidation across a period of sleep, but not across wakefulness. To the contrary, we found that expectation of a future test enhanced memory for both spatial and motor learning, but that this effect was equivalent across both wake and sleep retention intervals. These observations differ from those of least two prior studies, and fail to support the hypothesis that the “future relevance” of learned material moderates its consolidation selectively during sleep.

## Introduction

Following encoding, memory undergoes a process of “consolidation”, during which initially labile memory traces are stabilized and reorganized over time [1,2]. A large body of evidence now suggests that sleep is an important time during which memory consolidation occurs [3–6]. However, new research is pursuing the hypothesis that sleep may not strengthen all memories equally [7–10]. Instead, information that is most important to the individual may be preferentially consolidated during sleep, while irrelevant information is forgotten. For example, sleep has been reported to selectively strengthen memories that are emotional [8,11,12], associated with a reward [10], or anticipated to be useful the following day [9,13]. These data have been interpreted as an indication that memory consolidation during sleep uses an “intelligent” algorithm allowing us to selectively retain the most useful memories [7].

Yet despite current excitement surrounding the hypothesis of “selective” memory processing during sleep, the evidence supporting this notion has been inconsistent. In the case of reward, for example, at least one study reported that pairing learned information with a monetary reward enhanced consolidation during a period of post-training sleep, but not during a period of wakefulness [10]. While follow-up studies have confirmed that reward value affects subsequent memory, several have reported either that this effect is not exclusive to sleep [14–16], or that reward *negatively* affects memory consolidation during sleep [17].

Evidence that sleep selectively enhances emotional aspects of memory has perhaps been more consistent. A number of studies now show that emotional objects within complex scenes are preferentially consolidated during sleep, at the expense of background information and neutral objects [8,18–20]. Post-training sleep, especially REM sleep [21,22], has meanwhile been reported to selectively enhance memory for emotional texts and pictures, even when participants are tested 4 years after initial encoding [11]. Even so, several studies using different types of emotional memory tasks have failed to see an increased memory benefit of sleep for emotional, relative to neutral information [23–25].

Another line of research has examined the effect of “future relevance” on memory consolidation. This work stems from recent advances in cognitive neuroscience suggesting that the function of human memory is not to remember the past *per se*, but rather to prepare us for the future [26]. In line with this hypothesis, the stated “future relevance” of learned information has been studied as a moderator of consolidation during sleep—at least two recent studies have reported that information that participants *are told that they will later be tested on* is selectively processed during sleep [9,13]. Waking retention intervals, on the other hand, are proposed to make no such distinction [9,13]. But conflicting findings are emerging in this area as well—one recent study of emotional memory found that the expectation of a future test selectively enhanced emotional memory across *wakefulness*, but had no effect over a sleep retention interval [20]. Here, we aimed to further explore the hypothesis that test expectation enhances memory consolidation during sleep, using one motor learning task originally employed for this purpose by Wilhelm et al. (2011), and extending it to a spatial learning task previously shown by our laboratory to benefit from post-training sleep [9,13].

The current study also examined the effect of “future relevance” on the incorporation of learning experiences into dream content. Our prior research established that memory consolidation during sleep is reflected in the conscious experience of dreaming. Research participants commonly dream of engaging, interactive laboratory learning tasks [27,28], and the extent to which recently learned information is incorporated into dreams predicts subsequent memory [29–31]. We hypothesized that, if information with relevance to the future is selectively processed in the sleeping brain, dreams might similarly be influenced by the processing of recent memories with particular relevance to the future.

Thus, in the current study, we anticipated *1)* to replicate previous reports that future-relevant information shows greater sleep-dependent enhancement than other memories and *2)* to find that information relevant to an individual’s future also shows the highest rates of incorporation into dreaming. To test these hypotheses, we trained participants on two learning tasks and then manipulated whether or not they were informed about a delayed memory test, which occurred either following either a night of sleep or a day of wakefulness. As detailed below, we failed to find support for either of these hypotheses, but did observe a robust effect of expectation on memory consolidation overall.

## Methods

### Participants

N = 100 participants between the ages of 18 and 30 (mean age = 20yrs ±2SD; 60% female) were recruited through advertisement from the undergraduate student populations of Furman University in Greenville, SC (n = 74) and from Boston College in Boston, MA (n = 26). This research was approved by the institutional review boards of Furman University, Greenville, SC, and Beth Israel Deaconess Medical Center, Boston, MA. Respondents were excluded from participation if they were taking medication that could interfere with normal sleep patterns (including antidepressants, stimulants, hypnotics, opiates, and anticholinergics), if they had no prior experience playing 3D-style video games [32], if they had been diagnosed with a sleep disorder or mental disorder (by self-report), and if they were not fluent in English. Participants were asked to keep a regular sleep schedule for the 3 nights prior to the study (no bedtimes before 10pm or after 2am), to refrain from drugs or alcohol the day prior to the study, and to refrain from consuming caffeine after 10 am on the day of the study. Pre-study sleep schedule was confirmed using a sleep log. Participants were compensated at a rate of $10/hr.

### Procedures

**Fig 1:**
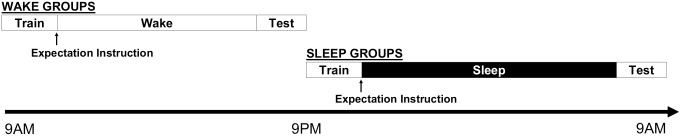
Experimental Design. After training on two learning tasks, participants were retested following an 11hr delay filled with either wakefulness or sleep. Immediately after encoding, *test expected* groups were informed that they would be later tested on the same material, whereas *test unexpected* groups were not.

All subjects were trained on both a spatial learning task and a motor learning task, in counterbalanced order (see below). Memory for both tasks was tested following an ≈11hr delay (Fig 1). We manipulated whether participants slept or remained awake during this retention interval, and whether or not participants were informed about the delayed memory test. Thus, participants were randomly assigned to one of four conditions: *wake + test expected (n = 18)*, *wake + test unexpected (n = 28)*, *sleep + test expected (n = 21)*, and *sleep + test unexpected (n = 30)*. Because we anticipated that some participants in the nominal “unexpected test” conditions would suspect the delayed test [9], participants were assigned to expectation groups at a 2:3 ratio, with a greater number of participants assigned to the *unexpected test* groups. As described below, this allowed us to follow the analysis methods of Wilhelm et al. (2011), reclassifying those who “suspected” the delayed retrieval test into a separate group.

Upon arrival at the laboratory, participants provided written informed consent before completing a demographics form, a 3-day sleep log, and the Epworth Sleepiness Scale (a measure of trait sleepiness [33]). Participants in the *wake* conditions trained on the learning tasks at 9:30am and then left the laboratory for the day, returning to be tested at 8:30pm. *Wake* participants were asked not to nap, and not to consume alcohol, caffeine, or other psychoactive substances during the day. Participants in the *sleep* conditions trained on the learning tasks at 9:30pm, spent the night in the sleep laboratory with polysomnographic (PSG) monitoring (see below), and then completed the delayed memory test at ≈8:30am. Just prior to each testing session, participants completed the Stanford Sleepiness Scale, a measure of state sleepiness [34].

Immediately following encoding, participants in the *test expected* groups were informed they would be retested on the exact same tasks during the second session. Those in the *test unexpected* groups were not informed they would be retested on these tasks. The exact instructions given were as follows:

Wake + Expected Test Group: “Ok, you’ve completed the first round of testing. However, when you return in the evening you will be doing the exact same maze and the exact same motor skills typing task. The maze will be the same maze as you saw just now, and the exit door will be in the same place. For the typing task, you will type the exact same sequence of numbers as quickly as possible.”
Wake + Unexpected Test Group: “Great, that’s all for these tests. When you return in the evening you will be completing some additional questionnaires and testing, which will include being asked about your daytime activities.”
Sleep + Expected Test Group: “Ok, you’ve completed the first round of testing. However, when you wake in the morning you will be doing the exact same maze and the exact same motor skills typing task. The maze will be the same maze as you saw just now, and the exit door will be in the same place. For the typing task, you will type the exact same sequence of numbers as quickly as possible.”
Sleep + Unexpected Test Group: “Great, that’s all for these tests. When you wake in the morning you will be completing some additional questionnaires and testing, which will include being asked some additional questions about your dreams.”

In the morning, participants completed an exit questionnaire that asked them the extent to which they had expected to be tested during the second sessions, with the options: “Yes-I pretty much knew I would be tested on the exact same thing,” “A little- I suspected I might be tested again on the same thing, but I wasn’t sure,” and “No-not at all. I didn’t at all realize I would be tested on the same thing again.”

Finally, participants completed a rehearsal questionnaire on which they used a 5-point scale to separately rate the frequency with which they had “thought about”, “imagined”, and “tried to remember” each of the two learning tasks during the retention interval.

#### Collection of Dream Reports

During the night, participants in the sleep groups were intermittently awoken to provide verbal reports of their mental experiences. These reports were subsequently scored as to whether they were related to pre-sleep training on the VMT. Reporting and scoring procedures followed the methods established in our prior work [28,35]. We have previously reported that this dream awakening procedure does not affect overnight consolidation of the VMT [35]. In brief, up to 10 “sleep onset” dream reports were collected during the first hour of the night, 30, 60 or 90 seconds after sleep onset (latencies following a randomized order). Three additional reports were collected during later the night: one from Stage 2 NREM sleep, one from REM sleep, and a final report upon awakening in the morning, regardless of sleep stage. At each of these time points, participants were awakened by calling their name, and instructed to verbally report “everything that was going through your mind” just before they were called. These reports were digitally recorded, transcribed, and scored for maze-related content by 2 independent judges, blind to experimental condition. Judges determined whether each report contained 1) content *directly* related to the VMT (100% rater agreement) or 2) content *indirectly* related to the VMT (99% rater agreement). A third judge (also blind to condition) provided tiebreak scores where raters disagreed.

#### Motor Sequence Typing Task (MST)

The Motor Sequence Typing task (MST) is a procedural learning task previously shown to benefit from post-training sleep [36], and which was employed by Wilhelm et al. in their 2011 study of the effect of future relevance on memory consolidation during sleep [9]. In this task, participants typed the sequence “4-1-3-2-4” as quickly and as accurately as possible using a computer keypad. At training, participants used their left hand to repeatedly type this sequence across 12 30sec trials, with 30sec breaks between each trial. In the test session 11hrs later, participants completed an additional 12 trials with the left hand. To test for generalization of motor learning to the opposite hand, participants then also completed 12 trials with the opposite (right) hand, thus retaining the same goal sequence, but completing this sequence using different muscle movements [37]). The primary dependent variable was the number of correct sequences typed per trial, which measures speed and accuracy of motor performance.

#### Virtual Maze Navigation Task (VMT)

Participants trained on a Virtual Maze Navigation Task (VMT) programmed using the Unreal game design platform (© Epic Games). Like the MST, memory for this task has previously been shown to benefit from post-training sleep [32,35]. In the VMT, participants are placed in a complex 3-dimensional maze environment and must navigate to the exit point across a series of trials as quickly and accurately as possible. The exit point is a white door, the location of which remains the same throughout the task. Participants used a keypad to move through the maze, which was displayed on a projector screen. Participants wore headphones, through which they heard the sound of running water, which increased in volume as they moved closer to the exit. To begin, participants were given 5min to explore the environment and become familiar with the layout of the maze in relation to the exit door. After exploration, participants were asked to find the exit door during 3 successive retrieval trials, with time limit of 10 minutes for each trial. If participants did not find the door within the 10 minutes, that trial was terminated and a new trial began. Each trial began from a different starting location in the maze, each approximately equidistant from the goal. During the testing session, participants performed 3 additional retrieval trials.

Three performance measures were calculated, following our prior work [35]. “Completion Time” is the time (seconds) taken to reach the exit on each trial. To analyze movement parameters during the task, the maze was overlaid with a 20x20 grid. “Distance Traveled” is the total number of grid-square boundaries crossed on each trial. “Backtracking” is then calculated as (1-# of unique grid squares entered/Distance Traveled), reflecting the proportion of a participant’s path that consists of re-entering previously entered squares.

On an exit questionnaire, participants selected which of 8 specific strategies they used to solve the maze. Four of the listed strategies were egocentric strategies (e.g. using landmarks, memorizing a series of left/right turns) and four were allocentric strategies (e.g. forming a mental map, imagining a bird’s eye view). For each participant, an “allocentric strategy score” was calculated as the ratio of allocentric strategies endorsed to egocentric strategies endorsed (Table 1).

**Table 1:** Experimental Group Characteristics at Baseline.

	Wake				Sleep				
	Unexpected Test		Expected Test		Unexpected Test		Expected Test		*p*
	Mean	±SD	Mean	±SD	Mean	±SD	Mean	±SD	
Age (yrs)	19.57	3.57	20.72	2.42	20.62	1.59	20.00	1.10	0.30
% Male	0.39	0.50	0.33	0.49	0.34	0.48	0.43	0.51	0.91
Game Experience	3.46	0.99	3.53	1.07	3.54	0.84	3.40	1.27	0.97
Pre-Study TST (min)	469.5	43.4	480.4	50.1	469.9	56.5	479.9	40.2	0.79
Epworth Score	8.39	3.02	8.28	3.21	7.66	3.57	8.24	3.46	0.84
SSS	2.52	0.94	2.67	1.09	3.34	1.26	3.24	1.00	0.02
Allocentric Score	1.82	0.82	1.72	0.96	1.93	0.96	2.10	0.97	0.61
VMT Time (sec)	230.8	180.8	248.9	178.7	230.9	156.8	212.4	174.1	0.95
MST Sequences	22.7	5.8	22.3	6.1	22.6	5.6	22.6	4.3	0.996

*Notes*. Video game experience was self-rated on 5-point scale; Pre-Study TST (total sleep time) = mins of sleep on the 3 nights prior to the experiment, as reported on the sleep log; Epworth Score = Epworth Sleepiness Scale (trait sleepiness); SSS = Stanford Sleepiness Scale (state sleepiness); Allocentric score = exit questionnaire ratio of allocentric strategies endorsed/egocentric strategies endorsed; VMT Time = Time to complete last training trial (trial 3); MST Sequences = mean # correct sequences typed on last 3 training trials; P-values are derived from a one-way ANOVA comparing across all four experimental groups.

#### Polysomnography (PSG)

PSG data were acquired with a standard montage of EEG (electroencephalography; locations F3, F4, C3, C4, O1, O2) referenced to the contralateral mastoids, electromyography (EMG) to monitor muscle tone, and electrooculography (EOG) to monitor eye movement (left and right outer canthus). Signals were recorded at 400Hz using a Grass-Telefactor AURA amplifier and Twin EEG & LTM Clinical Software (© Grass Technologies).

### Data Analysis

#### Exclusions

Data from 1 participant were excluded from analysis for failure to follow instructions in completing the learning tasks. Data from 2 participants were excluded from analysis because of extreme outlying scores on the VMT and/or MST (>3 interquartile ranges above or below the median). Data from n = 22 participants were selectively excluded from analyses of VMT performance, either because they failed to complete the task due to signs of possible “cybersickness” (a commonly induced symptom when moving through a virtual environment [38]), or because of technical problems with the task. Data from n = 3 participants were selectively excluded from analyses of MST performance due to technical problems with the task.

#### Sleep Data Analysis

30sec PSG epochs were manually scored for sleep stages according to the standard criteria published by the American Academy of Sleep Medicine [39]. The recordings for each participant were exported to BrainVision Analyzer 2.0 (© Brain Products) for further analysis. Following manual artifact rejection, a previously validated wavelet-based algorithm was applied to detect each sleep spindle oscillation during stage 2 sleep, separately at each EEG electrode [40,41].

#### Statistical Analysis

The effects of sleep and expectation on test performance were examined using 2(sleep vs. wake) x 2(expected vs. unexpected) ANCOVA models, controlling for baseline performance at the end of training. For the VMT, test performance was defined as the mean score across all 3 trials of the delayed test, controlling for performance on the last training trial (trial 3). For the MST, test performance was defined as the mean # of correct sequences typed on the first 3 trials of the delayed test, controlling for the mean # of correct sequences typed on the last 3 training trials.

## Results

### Expectation Manipulation Check

Instructing participants that they would later be tested significantly modulated expectation responses (χ^2^ (2, *N* = 95) = 12.78, *p* = .002), such that those randomly assigned to the *expected test* groups were much more likely to report on the exit questionnaire that they had expected or suspected the delayed retrieval test (χ^2^ (1, *N* = 95) = 6.90, *p* = .009).

### Expectation Instructions Enhanced Consolidation across both Sleep and Wake

**Fig 2:**
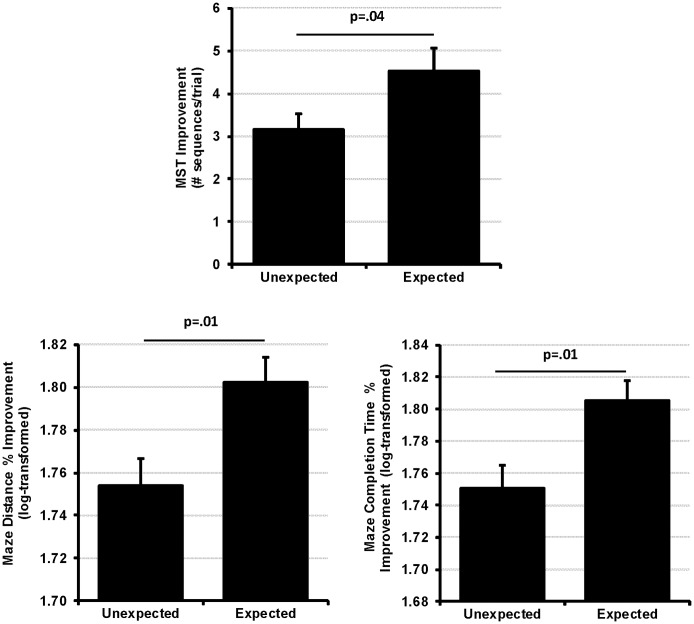
Effect of Expectation on Consolidation. The instruction to expect a future test enhanced performance on both the VMT and MST tasks. Error bars ±SEM. For clarity, this figure displays improvement from baseline. Statistical tests utilized an ANCOVA controlling for baseline performance.

Randomly assigned expectation instruction enhanced memory at delayed test for both the VMT and MST (Fig 2). Expectation significantly affected both Completion Time (main effect of expectation: *F*_1,71_ = 7.35, *p* = .008, η^2^_p =_ 0.09) and Distance Traveled to reach the goal (main effect of expectation: *F*_1,71_ = 6.85, *p* = .01, η^2^_p =_ 0.09) for the VMT, and showed a marginal impact on Backtracking (main effect of expectation: *F*_1,71_ = 3.43, *p* = .07, η^2^_p =_ 0.05). Similarly, for the MST, the number of correctly typed sequences was significantly greater in the *expected test* groups than the *unexpected test* groups (main effect of expectation: *F*_1,89_ = 4.18, *p* = .04, η^2^_p =_ 0.05).

**Fig 3:**
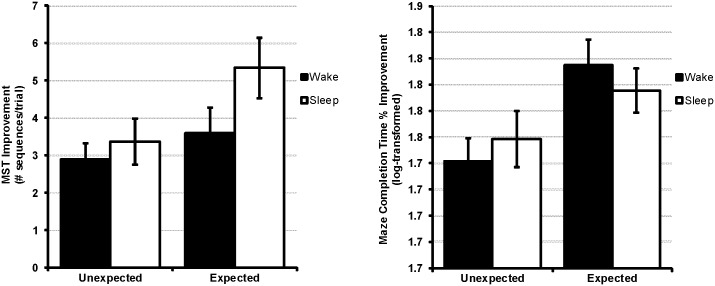
Expectation Equivalently Affects Memory across Both Wake and Sleep. The effect of expectation on memory consolidation was expressed equally across wake and sleep delays. Error bars ±SEM. For clarity, this Fig displays improvement from baseline. Statistical tests utilized an ANCOVA controlling for baseline performance.

Contrary to our hypotheses, the effect of test expectation was equivalent across sleep and waking retention intervals. The critical sleep x expectation interaction effect, testing for a selective effect of expectation on consolidation during sleep, did not approach statistical significance for any dependent measure on either learning task (VMT Completion Time: *F*_1,71_ = 0.77, *p* = .38; VMT Distance Traveled: *F*_1,71_ = 0.55, *p* = .46; VMT Backtracking: *F*_1,71_ = 0.0003, *p* = .99; MST correct sequences: *F*_1,89_ = 0.96, *p* = .33). Thus, as illustrated in Fig 3, the effect of expectation was nearly identical in wake and sleep subjects.

There were no significant main effects of sleep on VMT performance (Completion Time: p = .31, Distance Traveled: p = .37, Backtracking p = .54). There was a trend for sleep to benefit MST performance (main effect of sleep: *F*_1,89_ = 2.90, *p* = .09, η^2^_p =_ 0.03).

### Effects of Self-Reported Expectation

Exit questionnaire self-reports of participants’ expectation did not perfectly reflect the instructions they were given. Most notably, a full 70% of participants assigned to the *unexpected test* groups reported that they at least “suspected” they might be tested again on the learning tasks. Thus, following the procedures of Wilhelm et al., we conducted an additional set of analyses in which participants were reclassified according to their subjective responses on the exit questionnaire. Participants in the *unexpected test* groups who reported that they “suspected” or “knew” about the delayed test were reclassified as a separate group who suspected the memory test. Using this classification, we again found significant or near-significant effects of expectation on delayed memory performance (VMT Completion Time: *F*_1,71_ = 3.92, *p* = .03, η^2^_p =_ 0.10; VMT Distance Traveled: *F*_1,71_ = 4.31, *p* = .02, η^2^_p =_ 0.11; VMT Backtracking: *F*_1,71_ = 2.33, *p* = .11, η^2^_p =_ 0.06; MST correct sequences: *F*_1,89_ = 2.78, *p* = .07, η^2^_p =_ 0.06). Classifying subjects by exit questionnaire response, the sleep x expectation interaction effect was statistically significant for VMT Distance Traveled (*F*_1,71_ = 4.35, *p* = .02, η^2^_p =_ 0.11), but contrary to our hypotheses, this interaction was driven by the fact that a benefit of sleep on performance was selectively apparent for the *unexpected test* participants. The sleep x expectation interaction effect did not reach statistical significance for any other dependent measure.

Classifying subjects by exit questionnaire response, we found stronger evidence for an overall benefit of sleep for memory performance. There was a significant main effect of sleep on VMT Distance Traveled (*F*_1,71_ = 5.99, *p* = .02, η^2^_p =_ 0.08) and Completion Time (*F*_1,71_ = 4.86, *p* = .03, η^2^_p =_ 0.07), with a trend toward a sleep main effect for MST sequences typed (*F*_1,89_ = 2.59, *p* = .11, η^2^_p =_ 0.03).

### Right Hand Transfer Performance

The effect of expectation on MST performance appeared to be specific to the trained (left) hand. Right-hand transfer performance was not affected by expectation (analysis by randomly-assigned group: p = .25; analysis by exit questionnaire response: p = .60) or by sleep (analysis by randomly-assigned group: p = .53; analysis by exit questionnaire response: p = .22). There were also no significant sleep x expectation interaction effects for the right hand transfer MST test (analysis by randomly-assigned group: p = .86; analysis by exit questionnaire response: p = .39).

### Sleep Architecture and Sleep Spindles

Randomly-assigned expectation group did not significantly affect any aspect of sleep architecture (Table 2), and did not affect the number or density of sleep spindles during the night. We also conducted exploratory analyses of the correlation between overnight task improvement (test score—training score) with sleep architecture and sleep spindles. No correlations survived Bonferroni correction for multiple comparisons (10 sleep architecture variables + spindle counts at 6 electrodes = 16 correlations for each performance outcome, setting the adjusted significance criterion to p = .003).

**Table 2:** Sleep Architecture.

	Unexpected Test		Expected Test		*p*
	Mean	±SD	Mean	±SD	
TST (min)	473.3	86.7	450.9	56.2	0.31
Stage 1 (min)	30.9	14.3	26.9	8.5	0.22‡
Stage 2 (min)	230.0	86.6	233.4	53.8	0.88
SWS (min)	123.2	26.9	111.9	29.6	0.17
REM (min)	89.1	27.3	78.7	28.9	0.20
WASO (min)	92.8	50.0	87.5	27.0	0.66

TST = total sleep time; SWS = slow wave sleep; REM = rapid eye movement sleep; WASO = wake time after sleep onset; P-values are derived from an independent samples t-test.

^‡^ = t-test for unequal variances.

### Effects on Dream Experience

Overall, 269 dream report awakenings were conducted in the *Sleep + Unexpected Test* group, and 195 were conducted in the *Sleep + Expected Test* group. In the *Unexpected Test* group, 76% of awakenings yielded a report containing at least some mental content. In the *Expected Test* group, 85% of awakenings yielded a report with at least some mental content. However, only a small proportion of these reports were judged to be related to the VMT: 4 participants in the *Sleep + Unexpected Test* group reported a total of 5 maze-related dreams and 2 participants in the *Sleep + Expected Test* group reported a total of 2 maze-related dreams. A chi-square test of independence showed that the number of participants who incorporated the VMT into at least one dream report was not affected by expectation instruction (p = .76). For both the *Expected* and *Unexpected* groups, overnight improvement on the VMT did not differ significantly between subjects who did and did not report dreaming about the maze (Mann-Whitney U Test p-values all > 0.7).

### Rehearsal Questionnaire

Test expectation significantly affected participants’ exit questionnaire ratings of the frequency with which they “thought about” the MST, such that those in the *expected test* groups reported thinking about the MST more frequently during the retention interval (*F*_1,79_ = 4.98, *p* = .03, η^2^_p =_ 0.06). This effect did not differ by sleep condition (sleep x expectation interaction: p>.6). There were no other effects of experimental condition on rehearsal questionnaire responses for thinking about, imagining, or trying to remember either learning task. Participant ratings of thinking about the MST during the retention interval were not correlated with their subsequent test performance (p>.4).

### Baseline Group Characteristics

The experimental groups were equivalent at baseline in terms of demographics, video game experience, pre-study sleep schedule, and trait sleepiness (Table 1). There were no group differences in strategies used to complete the VMT (as reported on the exit questionnaire; Table 1). Although participants in the wake groups reported being moderately more sleepy at encoding relative to sleep subjects, scores on the Stanford Sleepiness Scale were not correlated with baseline performance on the VMT (Completion Time: p>.7, Distance Traveled: p>.6, Backtracking: p>.6) or the MST (p>.3).

## Discussion

Here, the perceived relevance of newly learned information impacted both motor and spatial memory across an 11hr retention interval. Expectation instructions were administered only after encoding was complete, and therefore must have influenced memory consolidation, rather than initial encoding. This study thus provides evidence that consolidation is subject to top-down modulation by our knowledge about the future usefulness of what we have just learned.

The observation that test expectation enhances memory is consistent with prior reports from the sleep literature [9,13], and also with related studies on the “intention superiority effect”. In the psychological literature, the “intention superiority effect” is a behavioral observation in which participants are faster in processing recently learned information that they believe they will use in the near future, relative to neutral information [42–44]. For example, in one task, participants learn a series of verbal “scripts” describing the steps involved in simple actions (e.g. making coffee or setting the table). Subjects are then informed that they will either actually perform these actions later, or that they will only observe someone else performing them. In a subsequent recognition task, participants respond more quickly to words that are part of to-be-performed scripts, as compared to to-be-observed scripts [44]. Our current observations extend these studies in several ways. First, the intention superiority effect has been described only immediately after the expectation manipulation takes place—here we demonstrate that this effect persists across at least 11hrs (as shown also in [9,13,20]). Second, although at least two prior studies have shown a similar effect of expectation on motor learning [9,42], the present study is the first to describe a similar effect for spatial memory, suggesting that the top-down influence of expectation on memory generalizes to a diverse array of memory systems.

The timeframe during which expectation exerted its effect on memory is unclear. Although memory enhancement was long-lasting, it could have been initiated immediately at the time of the instruction, via an influence on the early stages of cellular-level memory consolidation, for example as might be predicted by the synaptic tag and capture hypothesis [45]. Alternatively, later phases of the consolidation process, perhaps involving an influence on systems-level consolidation, could also have contributed.

Yet contrary to our hypothesis, we found no evidence that expectation *selectively* enhances consolidation across a period of sleep. Quite the opposite, there was a clear effect of expectation across both wakefulness and sleep. Our observations thus contradict those of at least two prior reports that test expectation selectively enhanced performance over sleep, but not across an equivalent period of wakefulness [9,13]. Our design was well-powered to detect a sleep x expectation interaction of the large magnitudes reported in these previous studies [9,13], yet this crucial interaction effect did not approach statistical significance for either learning task.

Although it is impossible to say for certain why our observations diverge from those reported by Wilhelm et al. [9] and van Dongen et al. [13], some explanations are more probable than others. We modeled the current study on Wilhelm et al. [9], using the same expectancy manipulation, the same general study design, one of the same learning tasks (the MST), and partially replicating their data analysis procedures. Still, this was not an exact replication and there are methodological differences that could explain the divergent outcome. For example, Wilhelm et al. conducted their research in Germany, the instructions were administered in German, the dream report collection aspect was not included, and the spatial learning task was not included. Wilhelm et al. also included a substantive cortisol sampling component to the study that may have provided a more effective “cover” for the importance of the morning procedures in the “unexpected” test groups. In theory, any of these factors might tap into an unrecognized moderating variable that somehow eliminates the sleep-selectivity of the test expectation effect.

However, our expectation manipulation was clearly effective in modulating both subjective reports of expectation and memory performance at delayed test, and *a priori*, we found no reason why any of these methodological differences should result in a failure to detect sleep-specific effects of expectation. Although the main effects of sleep on memory performance were weak in the current study, this cannot explain our failure to replicate prior observations of selectivity—there was a clear effect of expectation across *wakefulness* that was not present in these prior studies [9,13] (note also that sleep has been shown to enhance performance on both the VMT [29,32,35] and MST [36,46,47] in several prior investigations).

The addition of the dream reporting procedures is also unlikely to have interfered with consolidation during sleep, as we have previously reported that the same dream awakening procedure used here does not interfere with memory consolidation of the virtual maze task across a night of sleep [35], and participants in the present study were able to obtain an average of 7.8hrs of sleep during the experimental night. Still, the repeated awakenings induced by the dream reporting procedure are a possible limitation of the study, considering that sleep fragmentation is thought to interfere with memory consolidation [48].

The notion that sleep is an “intelligent” process that selectively enhances our most important memories is currently a popular narrative in sleep research [7]. Yet the current study is not the first investigation that has failed to support this hypothesis. As described above, one other recent study also reported that test expectation enhances memory consolidation across a 12hr interval of wake [20], and several studies have recently failed to support the related hypothesis that memories associated with a high reward value are preferentially enhanced by post-training sleep [14,15,17].

Notably, emotional memory studies have more consistently demonstrated selective memory processing during sleep, and in these studies, emotional salience is necessarily present at the time of encoding [8,11,19]. Although here we focused on the effect of a post-encoding manipulation, it is possible that salience manipulations during encoding are more reliably effective in driving consolidation during subsequent sleep.

Taken together, we conclude that methodological variation is not the most parsimonious explanation for these seemingly inconsistent results across studies. Importantly, we do not claim that Wilhelm et al. [9] and van Dongen et al.’s [13] reports of selective sleep-dependent consolidation are false-positives. The present study is unable to rule out the existence of a small-size expectation x sleep interaction effect (9,13). However, we do suggest that the variability in outcomes across studies is more likely explained by sampling error and measurement error, rather than by an unidentified moderating variable (see [49] for an excellent review of the surprisingly large variability in study outcomes that can be induced by modest sampling and measurement error). Considering the current state of conflicting results in the literature, the hypothesis that sleep selectively consolidates future-relevant memories should be approached cautiously as an interesting question that remains unsettled. Well-powered exact replications and meta-analyses of the existing studies would be two clear avenues to resolving these questions.

Finally, contrary to our hypothesis, expectation of a future test did not detectably increase the probability that participants would incorporate the maze navigation task into their dream reports. The low overall rate at which this task was incorporated into dreaming could have interfered with our ability to detect an effect of expectation on this measure.

In summary, we confirm prior reports that post-encoding information about “future relevance” influences the fate of new memories [9,13,42,43,50]. This effect appears to extend to a wide variety of memory types (verbal, motor, spatial), and persists across at least 11hrs. It remains unclear whether “future relevance” affects consolidation more strongly during a particular behavioral state (sleep vs. wakefulness). Yet regardless of the state(s) in which this occurs, it is evident that post-encoding memory processes are subject to a top-down influence of knowledge about the utility of new information. As in many areas of psychological research, future direct replication efforts will offer a path to resolving the apparent conflicts in this literature.
